# Methylglyoxal induces cell death through endoplasmic reticulum stress‐associated ROS production and mitochondrial dysfunction

**DOI:** 10.1111/jcmm.12893

**Published:** 2016-06-16

**Authors:** Chi‐Ming Chan, Duen‐Yi Huang, Yi‐Pin Huang, Shu‐Hao Hsu, Lan‐Ya Kang, Chung‐Min Shen, Wan‐Wan Lin

**Affiliations:** ^1^ Department of PharmacologyCollege of MedicineNational Taiwan UniversityTaipeiTaiwan; ^2^ Department of OphthalmologyCardinal Tien HospitalNew Taipei CityTaiwan; ^3^ School of MedicineFu Jen Catholic UniversityNew Taipei CityTaiwan; ^4^ Medical Research CenterCardinal Tien HospitalNew Taipei CityTaiwan; ^5^ Department of PediatricsCathay General HospitalTaipeiTaiwan; ^6^ Graduate Institute of Medical SciencesTaipei Medical UniversityTaipeiTaiwan

**Keywords:** methylglyoxal, ER stress, retinal pigment epithelium, mitochondria, reactive oxygen species, intracellular calcium

## Abstract

Diabetic retinopathy (DR) and age‐related macular degeneration (AMD) are two important leading causes of acquired blindness in developed countries. As accumulation of advanced glycation end products (AGEs) in retinal pigment epithelial (RPE) cells plays an important role in both DR and AMD, and the methylglyoxal (MGO) within the AGEs exerts irreversible effects on protein structure and function, it is crucial to understand the underlying mechanism of MGO‐induced RPE cell death. Using ARPE‐19 as the cell model, this study revealed that MGO induces RPE cell death through a caspase‐independent manner, which relying on reactive oxygen species (ROS) formation, mitochondrial membrane potential (MMP) loss, intracellular calcium elevation and endoplasmic reticulum (ER) stress response. Suppression of ROS generation can reverse the MGO‐induced ROS production, MMP loss, intracellular calcium increase and cell death. Moreover, store‐operated calcium channel inhibitors MRS1845 and YM‐58483, but not the inositol 1,4,5‐trisphosphate (IP3) receptor inhibitor xestospongin C, can block MGO‐induced ROS production, MMP loss and sustained intracellular calcium increase in ARPE‐19 cells. Lastly, inhibition of ER stress by salubrinal and 4‐PBA can reduce the MGO‐induced intracellular events and cell death. Therefore, our data indicate that MGO can decrease RPE cell viability, resulting from the ER stress‐dependent intracellular ROS production, MMP loss and increased intracellular calcium increase. As MGO is one of the components of drusen in AMD and is the AGEs adduct in DR, this study could provide a valuable insight into the molecular pathogenesis and therapeutic intervention of AMD and DR.

## Introduction

The retinal pigment epithelium (RPE) is a component of the outer blood–retinal barrier (BRB) and carries out several important functions for the maintenance of the visual system. RPE cells are believed to play a crucial role for the pathogenesis of diabetic retinopathy (DR) 1 and age‐related macular degeneration (AMD) 2. DR is a common complication of diabetes mellitus (DM) and is the leading cause of acquired blindness in people aged 20–79 years 3. On the other hand, AMD is the most common cause of visual impairment in individuals over the age of 55 years in developed countries 4 with characterized accumulation of lipid‐ and protein‐rich deposits under the aged RPE that renders oxidant injury and heralds the onset of early AMD 5. Much evidence indicates that hyperglycaemia is not only a primary factor in the development of DR, but also an important contributor to AMD 6. Chronic exposure of the retina to hyperglycaemia gives rise to accumulation of advanced glycation end products (AGEs) in RPE basement membrane that plays an important role in both DR 7 and AMD 6, 8. AGEs formation is a natural function of ageing, and AGEs act as mediators of neurodegeneration during ageing and AMD. AGEs‐modified proteins that are highly formed in diabetic patients are also components of drusen, the sub‐RPE deposits that confer increased risk of AMD onset 9. Moreover, the receptor for AGEs (RAGE) is ubiquitously expressed in various retinal cells and is up‐regulated in the retinas of diabetic patients 10 and in RPE cells associated with basal deposits occurring in AMD 11.

Methylglyoxal (MGO) is formed by various biochemical pathways and is present under normal physiological conditions in all biological systems 12. Under glycolysis, MGO arises from two intermediates, glyceraldehyde phosphate and dihydroxyacetone phosphate by non‐enzymatic phosphate elimination 13, 14. Moreover, MGO may form through lipid peroxidation and from an intermediate of threonine catabolism, 3‐aminoacetone 14. The MGO within AGE causes irreversible effects on protein structure and function, associated with misfolding 15, and leading to cell apoptosis in various cell types 16. Moreover, MGO is released upon photodegradation of bisretinoids, A2E and all‐*trans*‐retinal dimer, which accumulate as lipofuscin in RPE 8. It can suppress proliferation of RPE cells and enhance autophagy flux 17. However, the RPE death mechanism related to endoplasmic reticulum (ER) stress that caused by MGO remains elusive.

The ER is a site for protein folding and maturation, as well as an intracellular location for Ca^2+^ store that plays a crucial role in signal transduction 18. ER stress triggers the unfolded protein response (UPR), which is distinguished by the action of three signalling proteins named inositol‐requiring 1 alpha (IRE1α)/spliced X‐box binding protein 1 (XBP1), double‐strand RNA activated kinase‐like ER kinase (PERK)/eukaryotic translation initiation factor 2 alpha (eIF2α)/activating transcription factor 4 (ATF4) and ATF6 19. CCAAT/enhancer‐binding protein homologous protein (CHOP) (also known as growth arrest and DNA‐damage‐inducible protein GADD153) is a pro‐apoptotic transcription factor associated exclusively with ER stress‐induced apoptosis 19. Many studies showed that ER stress is associated with DR 20, 21 and AMD 22. MGO has been shown to induce ER stress in lens epithelial cells 23. However, the link between MGO‐induced ER stress and cell death remains unknown. As MGO is highly related to DR and AMD, it is crucial to understand the underlying mechanism of causing RPE cell death. In this study, we found MGO can induce ARPE‐19 cell death through an active oxygen species (ROS)‐dependent manner, involving mitochondrial membrane potential (MMP) loss, intracellular calcium increase and ER stress response. Moreover, store‐operated calcium (SOC) channel inhibitor and ER chaperon can block the MGO‐induced ROS production, MMP loss and intracellular calcium increase in ARPE‐19 cells.

## Materials and methods

### Materials

Methylglyoxal, 3‐(4,5‐dimethylthiazol‐2‐yl)‐2,5‐diphenyltetrazolium bromide (MTT), N‐acetylcysteine (NAC), 4‐phenyl butyric acid (4‐PBA), MRS1845, YM‐58483, caffeine, dichlorodihydrofluorescein diacetate (H_2_DCFDA), dihydroethidium (DHE) and propidium iodide (PI) were obtained from Sigma‐Aldrich Co (St Louis, MO, USA). Xestospongin C was purchased from Tocris Bioscience (Bristol, UK). Z‐Val‐Ala‐Asp‐fluoromethylketone (z‐VAD‐FMK), salubrinal and 1,2‐bis(2‐aminophenoxy)ethane‐N,N,N′,N′‐tetraacetic acid tetrakis/acetoxymethyl ester (BAPTA/AM) were purchased from Calbiochem (Darmstadt, Germany). The antibodies specific for caspase‐3, caspase‐9, eIF2α, phospho‐eIF2α and poly(ADP‐ribose) polymerase 1 (PARP1) were purchased from Cell Signalling Technology (Beverly, MA, USA). The antibodies specific for glucose‐regulated protein 78 (GRP78), CHOP, PERK, phospho‐PERK and β‐actin were purchased from Santa Cruz Biotechnology (Santa Cruz, CA, USA). The antibodies specific for ATF4, ATF6, IRE1β and XBP1 were purchased from Abcam (Cambridge, UK).

### Cell cultures

Adult human RPE cell line ARPE19 was purchased from Food Industry Research and Development Institute (Hsinchu, Taiwan). These cells were maintained in Dulbecco's Modified Eagle's Medium/Nutrient Mixture F‐12 (DMEM/F12) supplemented with 10% foetal calf serum (GibcoBRL, Invitrogen Life Technologies, Carlsbad, CA, USA), 100 units/ml penicillin and 100 μg/ml streptomycin (Sigma‐Aldrich Co.). The cells were cultured in a humidified incubator at 37°C and 5% CO_2_. For most of the experiments, cells reaching a 90–95% of confluence were starved and synchronized in serum‐free DMEM for 24 h before they were subjected to further analysis.

### Measurement of cell viability by MTT assay

Cells (10^4^/ml) plated in 96‐well plates were incubated with the indicated drugs at 37°C. MTT (5 mg/ml) was added for 45 min, then the culture medium was removed, and the formazan granules generated by live cells were dissolved in 100% DMSO and shaken for 10 min. The optical densities (ODs) at 550 and 630 nm were measured using a microplate reader. The net absorbance (OD550–OD630) indicates the enzymatic activity of mitochondria and implies the cell viability.

### Flow cytometric annexin V‐FITC/PI assay

The cell surface exposure of phosphatidylserine and the plasma membrane impairment of cells were assessed using annexin V–FITC Apoptosis Detection Kit (Calbiochem). Briefly, suspension of treated/control ARPE‐19 cells, containing 5 × 10^5^ cells, was washed with PBS and resuspended in 0.5 ml cold binding buffer. Then, 1.25 μl of annexin V‐FITC was added and the cells were incubated in the dark for 15 min at room temperature. Following incubation, the cells were centrifuged at 100 × *g* for 5 min and the supernatant was removed. The cell pellet was resuspended in 0.5 ml cold binding buffer, and 10 μl of the 30 μg/ml PI solution was added. Cell samples were placed on ice, away from light, and FITC and PI fluorescence was immediately measured by using flow cytometer (Cytomics FC500; Beckman‐Coulter, Brea, CA, USA). Data were analysed using CellQuest Pro software (Becton Dickinson, Franklin Lakes, NJ, USA). The populations of live cells, early apoptotic cells, late apoptotic and necrotic cells were determined.

### Flow cytometric PI uptake assay

The cell membrane integrity was determined by the ability of cells to take up PI. After trypsinization, cells were collected by centrifugation, washed once with PBS, and resuspended in PBS containing 25 μg/ml PI for 20 min at 37°C. Then, the cells were measured by using flow cytometer (Cytomics FC500; Beckman‐Coulter). Data were evaluated using CellQuest Pro software (Becton Dickinson). The level of PI taken up by the cells was measured and represented as the percentage of control.

### Determination of the cytosolic ROS

Intracellular ROS production was detected using H_2_DCFDA and DHE for H_2_O_2_ and O_2_^−^ respectively. After drug treatment, ARPE‐19 cells were washed with PBS and incubated with 10 μM H_2_DCFDA or 5 μM DHE at 37°C for 30 min. Subsequently, the cells were washed in PBS, trypsinized and the fluorescence intensity was measured by flow cytometry (Cytomics FC500; Beckman‐Coulter) at excitation/emission wavelengths of 485/530 nm and 488/512 nm for H_2_O_2_ and O_2_^−^ respectively. For each sample, H_2_O_2_ or O_2_^−^ production was expressed as mean fluorescence ratio (fluorescence of exposed cells/fluorescence of control cells) from the same experiment.

### Determination of the mitochondrial membrane potential (MMP)

Rhodamine 123 is a fluorescent cationic dye that binds to polarized mitochondrial membrane and accumulates as aggregates in the mitochondria of normal cells. ARPE‐19 cells were cultured in the absence or presence of MGO for 6 h, and then incubated with 1 μM rhodamine 123 for 30 min. The cells were then centrifuged and resuspended in PBS. Changes in the MMP were detected by flow cytometry (Cytomics FC500; Beckman‐Coulter).

### Intracellular calcium measurement

Intracellular calcium was measured by Fluo‐3. After treating cells with the indicated drugs for different time periods, cells were incubated in PBS containing Fluo‐3 (3 μM) for 30 min at 37°C. Cells were subjected to a flow analysis by flow cytometry (Cytomics FC500; Beckman‐Coulter).

### Cell lysate preparation and Western blot analysis

After stimulation, the medium was aspirated. Cells were rinsed twice with ice‐cold PBS, and 25–100 μl of cell lysis buffer (20 mM Tris–HCl, pH 7.5, 125 mM NaCl, 1% Triton X‐100, 1 mM MgCl_2_, 25 mM β‐glycerophosphate, 50 mM NaF, 100 μM Na_3_VO_4_, 1 mM PMSF, 10 μg/ml leupeptin and 10 μg/ml aprotinin) was then added to each well. After harvesting, cell lysates were sonicated and centrifuged, and equal protein amounts of soluble protein, as determined by the Bradford protein assay, were denatured, subjected to sodium dodecylsulfate polyacrylamide gel electrophoresis (SDS‐PAGE), and transferred to a polyvinylidene difluoride membrane. Non‐specific binding was blocked with TBST (50 mM Tris–HCl, pH 7.5, 150 mM NaCl and 0.02% Tween 20) containing 5% non‐fat milk for 1 h at room temperature. After immunoblotting with the first specific antibody, membranes were washed three times with TBST and incubated with a horseradish peroxidase (HRP)‐conjugated secondary antibody for 1 h. The dilution folds of first specific antibodies were 1:1000 and β‐actin was 1:10,000. After three washes with TBST, the protein bands were detected with enhanced chemiluminescence detection reagent. To make sure equal amounts of sample protein were applied for electrophoresis and immunoblotting, β‐actin was used as an internal control.

### Statistical analysis

All data were obtained from at least three separate experiments and presented as mean ± standard error (SE). Analysis of variance was used to assess the statistical significance of the differences. A *P* value of less than 0.05 was considered statistically significant.

## Results

### Methylglyoxal induces a mixed type of cell death in ARPE‐19 cells

**Figure 1:**
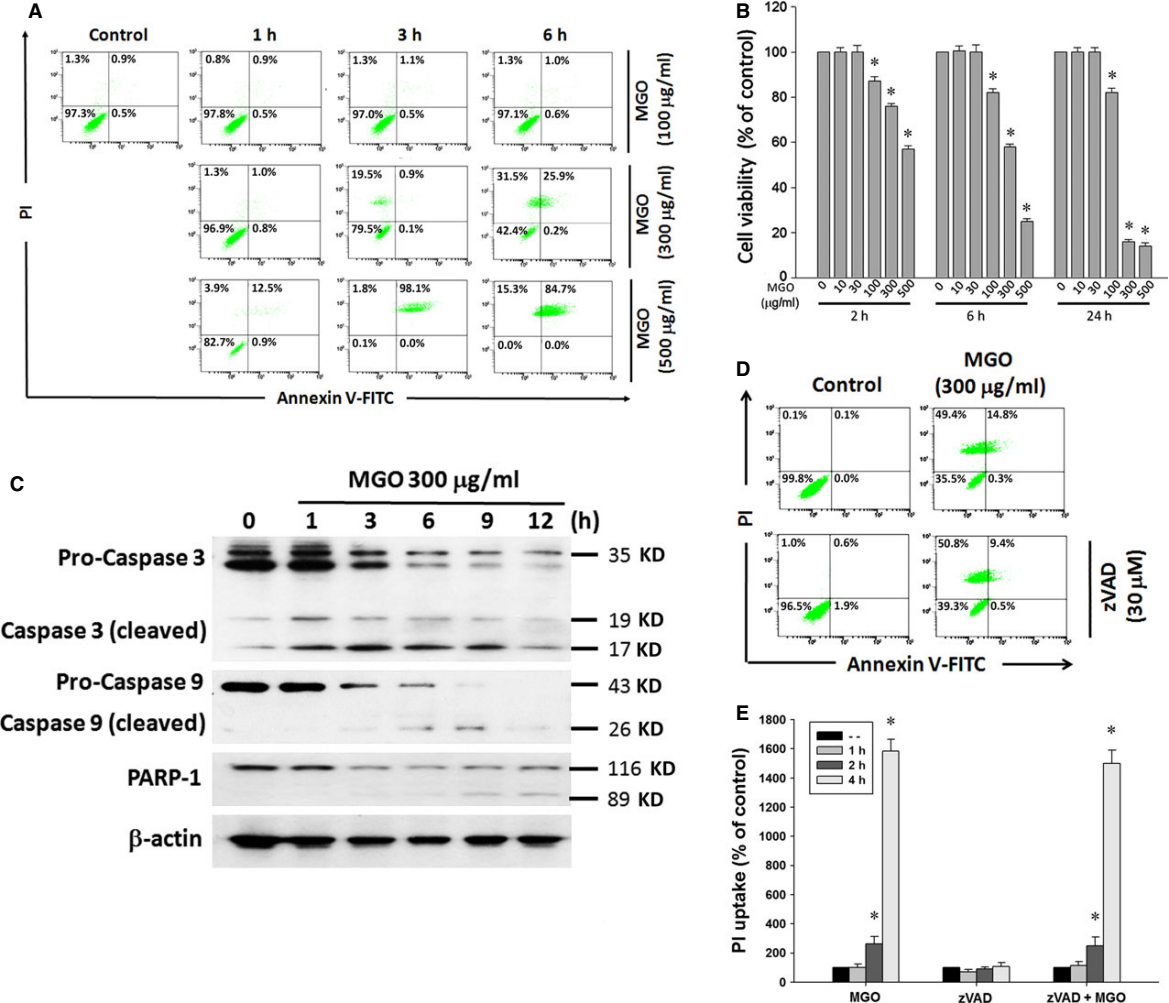
MGO decreases cell viability and induces caspase‐independent cell death in ARPE‐19 cells. (**A**) Cells were treated with various concentrations (100, 300 or 500 μg/ml) of MGO for 1, 3 or 6 h, and then stained with annexin V and PI, and evaluated by flow cytometry. (**B**) Cells were treated with various concentrations (10–500 μg/ml) of MGO for 2, 6 or 24 h. Cell viability was determined by MTT assay and the percentages of viability as compared to vehicle‐treated cells were plotted as the mean ± SE of at least three independent experiments. (**C**) Cells were treated with 300 μg/ml MGO for the indicated times. Cells were lysed and expression levels of indicated proteins were detected by Western blotting by using antibodies against caspase 3, caspase 9, PARP‐1 and β‐actin. (**D**) Cells were treated with pan‐caspase inhibitor zVAD (30 μM) for 30 min, followed by the treatment with 300 μg/ml MGO for 6 h. Cell viability was determined by annexin V/PI double staining assay. (**E**) After cells were treated with zVAD and MGO (300 μg/ml) for 1, 2 and 4 h, PI uptake was measured by flow cytometry. *P < 0.05, indicating the significant induction of cell death by MGO.

To understand the effect of MGO on cell viability, ARPE‐19 cells were treated with 100, 300 or 500 μg/ml MGO for 1, 3 and 6 h followed by an annexin V‐FITC/PI double labelling assay. The percentages of viable (annexin V‐negative/PI‐negative), early apoptotic (annexin V‐positive/PI‐negative), late apoptotic (annexin V‐positive/PI‐positive) and necrotic cells (annexin V‐negative/PI‐positive) were determined. Under the 100 μg/ml MGO treatment for 6 h, the cell death is moderate. However, increasing concentration to 300 μg/ml, the percentage of viable cells within 6 h incubation was significantly decreased from 97.3% to 42.4%, the portion of late apoptotic cells was significantly increased from 0.9% to 25.9% and the fraction of necrotic cells was increased from 1.3% to 31.5% at 6 h (Fig. 1A). At higher concentration of MGO (500 μg/ml), there was no viable cells left after 6 h incubation, and the portion of late apoptotic cells was rapidly increased in 1 h incubation, much faster than the onset time (6 h) for 300 μg/ml. The results suggest that MGO at concentrations tested induces an early necrotic cell death, which is followed by the exposure of the apoptotic marker phosphatidylserine. MGO‐induced ARPE‐19 cells death was further confirmed by the MTT assay after treatment with MGO (10–500 μg/ml) for 2, 6 and 24 h. Data revealed that MGO reduced cell viability in a concentration‐dependent manner and the cell viability was reduced by 40% at 300 μg/ml MGO in 6 h (Fig. 1B).

Since caspase activation is one of the mechanisms of apoptotic process, we examined the effect of MGO on caspase activation. ARPE‐19 cells were treated with 300 μg/ml MGO and cleavage of caspase‐9, ‐3 and PARP1 were detected by Western blot analysis. The results showed a gradual increase of the cleaved fragments of caspase‐9, ‐3 and PARP1 by MGO treatment for 12 h (Fig. 1C). To determine whether caspase is a crucial factor in MGO‐induced apoptosis in ARPE‐19 cells, cells were pre‐treated with pan‐caspase inhibitor zVAD for 1 h, followed by treatment with 300 μg/ml MGO for 6 h. Cell viability by annexin V‐FITC/PI double labelling assay showed that viable cells were significantly decreased at 6 h, but this portion of viable cells cannot be reversed by adding zVAD (Fig. 1D), indicating MGO induces a caspase‐independent mixed type of cell death. Consistently, data of PI uptake revealed that MGO‐induced cell necrosis was not altered by zVAD (Fig. 1E).

### Increased mitochondrial ROS production contributes to mitochondrial membrane potential loss and cell death caused by MGO

**Figure 2:**
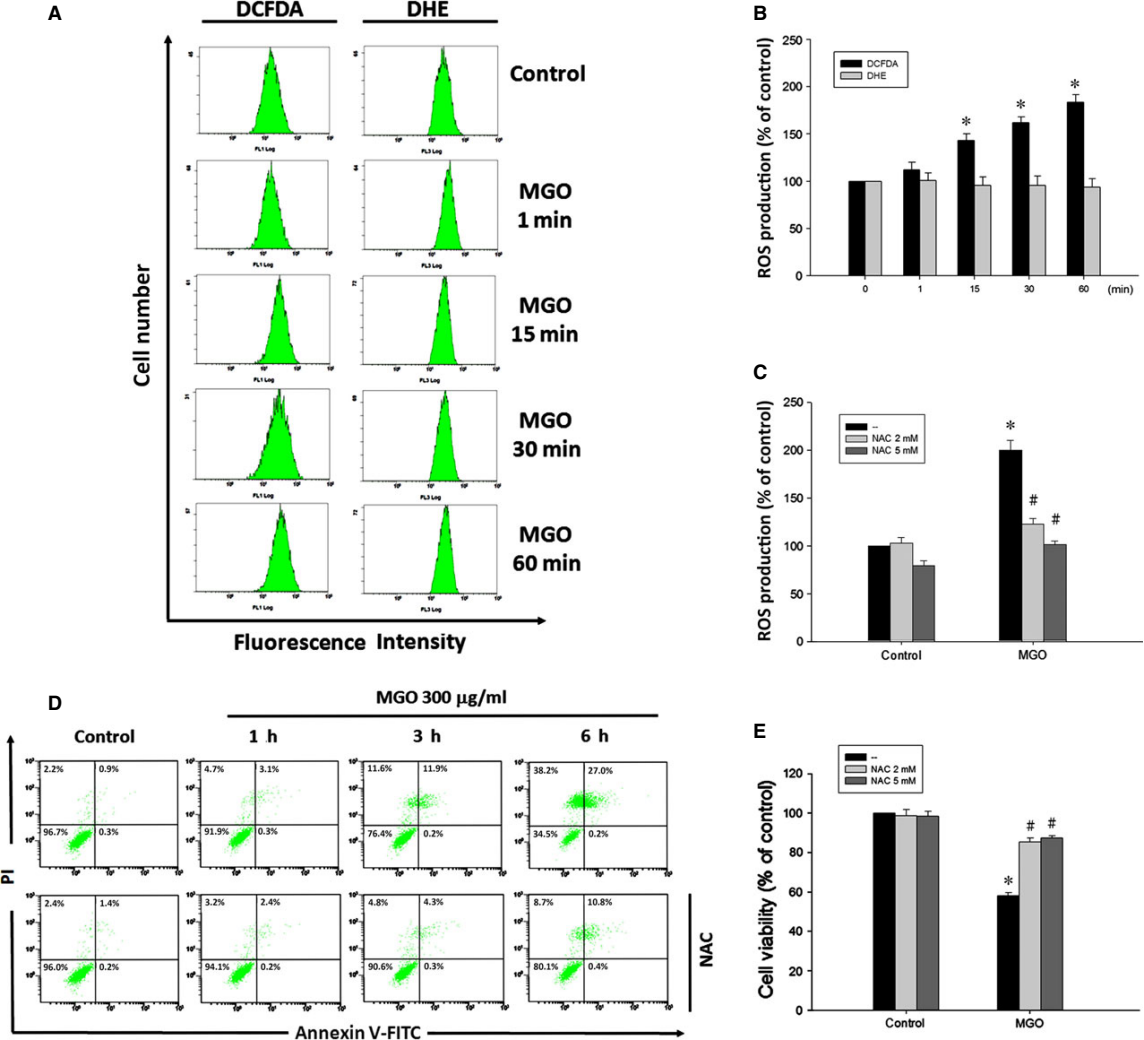
ROS scavenger suppresses MGO‐induced ROS generation and cell death in ARPE‐19 cells. (**A, B**) Cells were incubated with 300 μg/ml MGO for the indicated times, and then treated with H_2_ DCFDA (10 μM) or DHE (5 μM) for 30 min. Cellular ROS levels were measured using flow cytometry, and presented as percentages of control. (**C**) After pre‐treatment with 2 or 5 mM NAC for 30 min, MGO‐induced mitochondrial ROS production at 1 h was measured with H_2_ DCFDA by using flow cytometry. (**D**) MGO‐induced cell death was assessed by pre‐treatment with NAC (2 mM) for 6 h. Cells were stained with annexin V and PI, and evaluated by flow cytometry. (**E**) Cell viability was determined by MTT assay at 6 h. *P < 0.05, indicating the significant increase of mitochondrial ROS production (**B, C**) and decrease of cell viability (**E**) by MGO. ^#^ *P* < 0.05, indicating the significant effects of NAC to reverse the actions of MGO in ROS production (**C**) and cell death (**E**).

To determine whether MGO could induce accumulation of ROS in ARPE‐19 cells, cells were treated with 300 μg/ml MGO for the indicated time within 1 h, and were then treated with H_2_DCFDA or DHE for 30 min. The data of flow cytometry indicated that H_2_O_2_ generation was rapidly increased by MGO at 15 min (47.5%) and kept increasing to 96.7% at 1 h. However, cellular O_2_^−^ was not increased (Fig. 2A and B). Pre‐treatment with 2 or 5 mM NAC abrogated MGO‐induced ROS production at 1 h (Fig. 2C). Concomitantly, NAC can reverse the MGO‐induced cell death as indexed by both the annexin V‐FITC/PI double labelling (Fig. 2D) and MTT assay (Fig. 2E). These results suggest the involvement of mitochondrial ROS in MGO‐induced cell death in ARPE‐19 cells.

**Figure 3:**
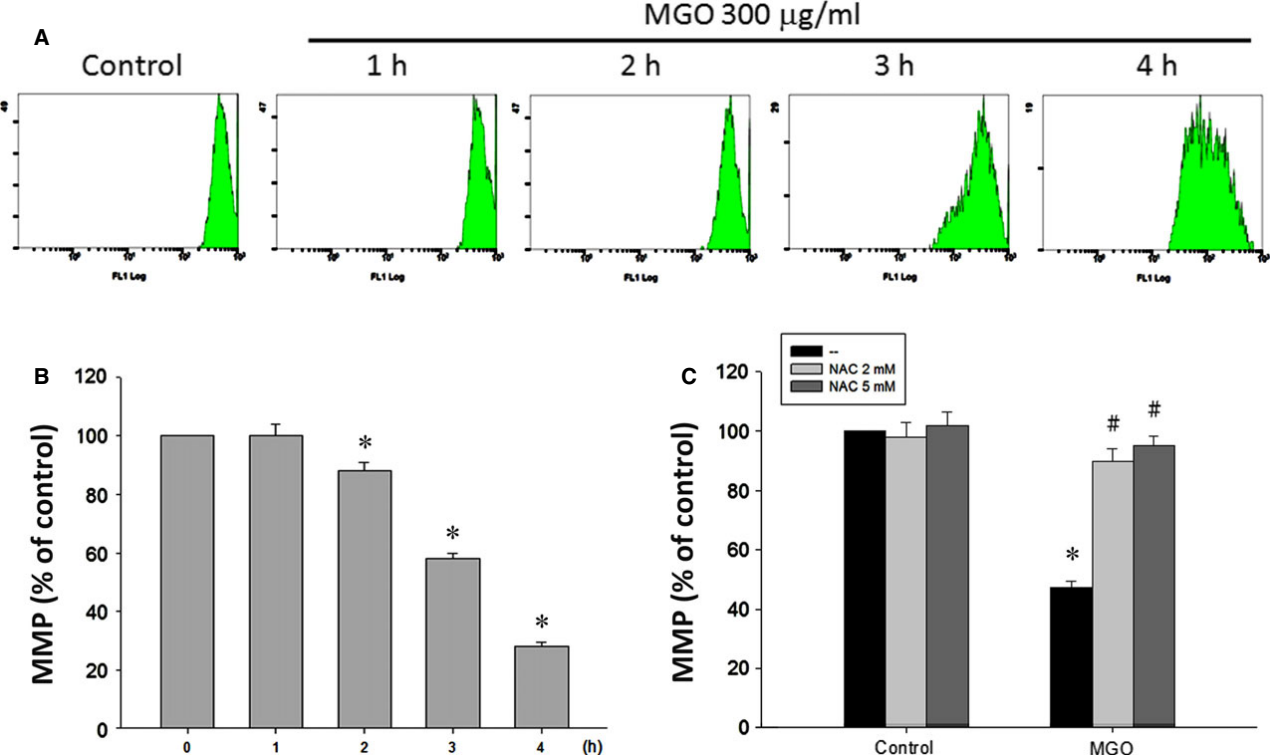
ROS scavenger suppresses MGO‐induced MMP reduction in ARPE‐19 cells. (**A, B**) Cells were incubated with 300 μg/ml MGO for 1, 2, 3 or 4 h. MMP was measured with rhodamine 123 by using flow cytometry. (**C**) Cells were pre‐incubated with 2 or 5 mM NAC for 30 min and then incubated with 300 μg/ml MGO for 3 h. *P < 0.05, indicating the significant decrease of MMP by MGO. ^#^ *P* < 0.05, indicating the significant ability of NAC to restore MMP under MGO treatment.

Since mitochondrial integrity is highly related to the cell viability, we determined whether MGO induces mitochondrial dysfunction by testing the MMP of the RPE cells. Cells were treated with 300 μg/ml MGO for the indicated time within 4 h, and the MMP was measured with rhodamine 123 by using flow cytometry. Results revealed the MMP loss from 1 to 4 h progressively (Fig. 3A and B), and this event was completely reversed by NAC (Fig. 3C). These results suggest that increased ROS contributes to the loss of MMP under MGO treatment in RPE cells.

### MGO increases intracellular calcium level through SOC pathway, and intracellular calcium and ROS exert an amplified effect to induce MMP loss

**Figure 4:**
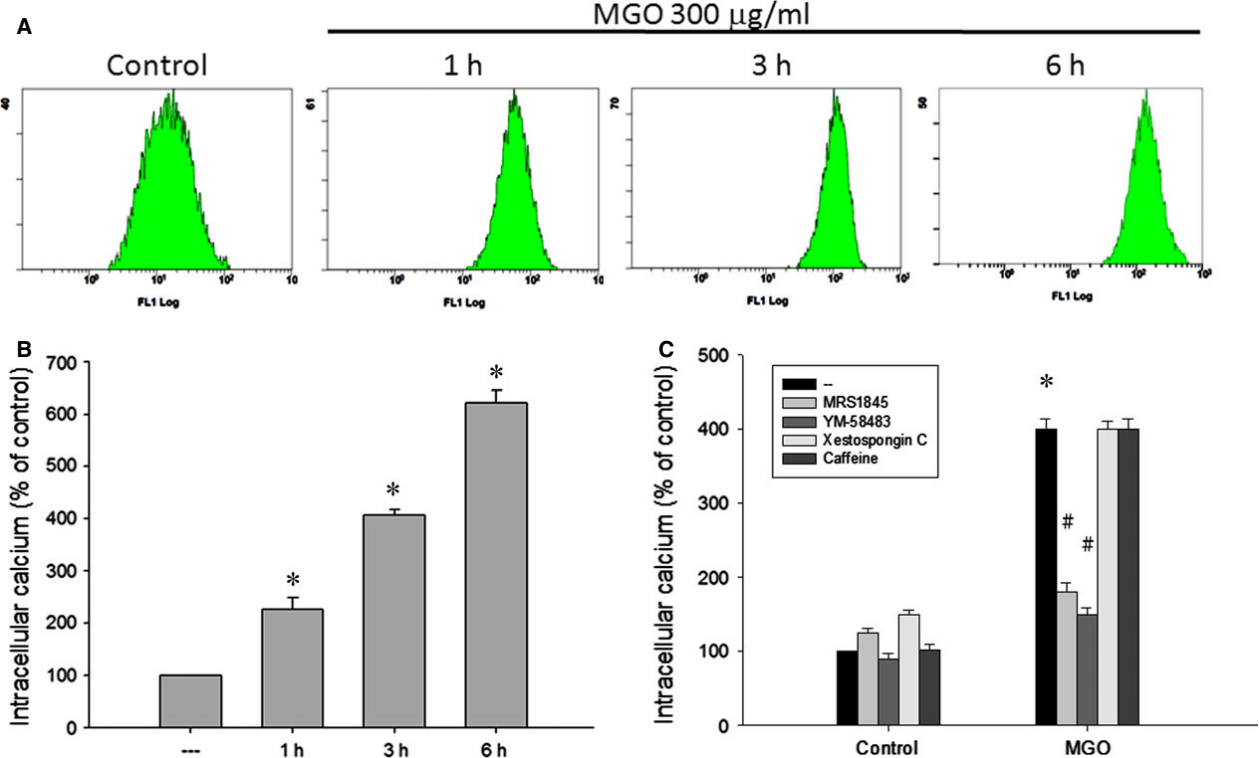
SOC channel inhibitors reduce MGO‐induced intracellular calcium increase in ARPE‐19 cells. (**A, B**) Cells were incubated with 300 μg/ml MGO for 1, 3 or 6 h. (**C**) Cells were pre‐treated with MRS1845 (10 μM), YM‐58483 (10 μM), xestospongin C (1 μM) or caffeine (10 mM) for 30 min, and then incubated with 300 μg/ml MGO for 3 h. Intracellular calcium levels were determined by using Fluo‐3 AM and flow cytometry. *P < 0.05, indicating the significant increase of intracellular calcium level by MGO. ^#^ *P* < 0.05, indicating the significant inhibition of MGO‐elicited intracellular calcium increase.

As calcium is a critical signal in the cell death pathway, we tested whether cytosolic calcium level is changed after MGO treatment. As a result, MGO can increase the intracellular calcium level in RPE cells after 1 h of incubation, and this increasing effect continued to 6 h by 6.3 folds (Fig. 4A and B). In order to understand the sources and pathway for intracellular calcium elevation, we examined the MGO‐induced intracellular calcium levels with the use of the SOC channel inhibitors, MRS1845 and YM‐58483 24, 25, 26, as well as the selective and membrane permeable IP_3_ receptor inhibitor xestospongin C 27, 28. We found that MRS1845 and YM‐58483 can significantly block the increase of the intracellular calcium, while xestospongin C did not have this effect (Fig. 4C). Moreover, caffeine, a ryanodine receptor activator 29 also failed to affect the increased intracellular calcium (Fig. 4C). These results suggest that MGO can induce extracellular calcium entry through SOC pathway.

**Figure 5:**
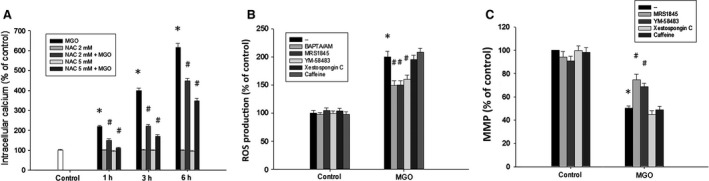
Reciprocal amplification of ROS production and intracellular calcium increase in MGO‐treated ARPE‐19 cells. (**A**) Cells were pre‐incubated with NAC (2 or 5 mM) for 30 min and then incubated with 300 μg/ml MGO for 1, 3 or 6 h. Intracellular calcium levels were determined by using Fluo‐3 AM and flow cytometry. (**B, C**) Cells were pre‐treated with BAPTA/AM (10 μM), MRS1845 (10 μM), YM‐58483 (10 μM), xestospongin C (1 μM) or caffeine (10 mM) for 30 min and then incubated with 300 μg/ml MGO. After 1 h, cellular ROS levels were measured with H_2_ DCFDA by using flow cytometry (**B**). After 3 h, MMP was measured with rhodamine 123 by using flow cytometry (**C**). *P < 0.05, indicating the significant effects of MGO. ^#^ *P* < 0.05, indicating the significant inhibition of MGO responses by indicated agents.

Afterwards, we examined the mutual interaction of the increased intracellular calcium and ROS under MGO stimulation. We found that NAC (2 and 5 mM) can reverse the MGO‐induced intracellular calcium increase in a concentration‐dependent manner (Fig. 5A). Conversely, calcium chelator BAPTA/AM and SOC channel inhibitors, MRS1845 and YM‐58483, can inhibit MGO‐induced ROS production at 1 h (Fig. 5B). In contrast, IP_3_ receptor inhibitor xestospongin C and aryanodine receptor activator caffeine cannot affect intracellular calcium increase caused by MGO (Fig. 5B). Consistently, MRS1845 and YM‐58483, but not xestospongin C and caffeine, can reverse the MGO‐induced MMP loss (Fig. 5C). These results altogether suggest the existence of an amplification effect of mitochondrial ROS production and SOC‐mediated calcium influx, and the coordinated effect to decrease MMP under MGO treatment.

### MGO‐induced ER stress response mediates ROS production, calcium increase and MMP loss

**Figure 6:**
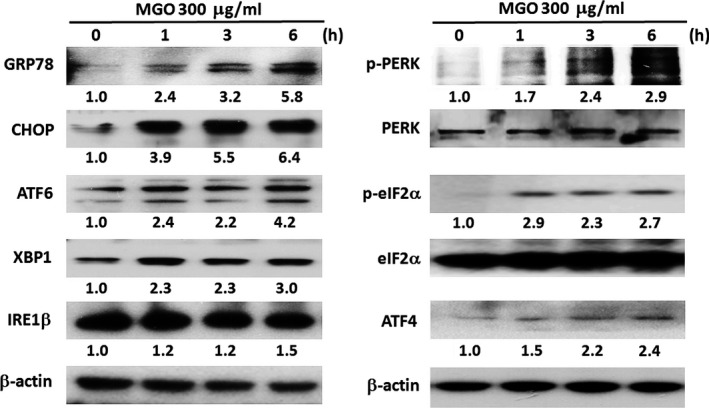
MGO induces ER stress response in ARPE‐19 cells. Cells were treated with 300 μg/ml MGO for the indicated times. Cell lysates were analysed by Western blot with antibodies specific to GRP78, CHOP, ATF6α (p90), XBP1, IRE1β, p‐PERK, PERK, p‐eIF2α, eIF2α, ATF4 and β‐actin. Results were representative of three independent experiments. The changes of protein expression were normalized to β‐actin (the cases of GRP78, CHOP, ATF6, XBP‐1, IRE1β and ATF4) or total protein (the cases for PERK and eIF2α), and then compared to the control group without MGO treatment.

As MGO can induce ER stress in lens epithelial cells 23, we determined whether MGO could also induce ER stress in RPE cells. First, we found the time‐dependent effects of MGO within 6 h to increase the protein expressions of GRP78, CHOP, ATF6 and ATF4, protein phosphorylation of eIF2α and PERK, and spliced XBP1and ATF6 formation (Fig. 6). These data suggest the ability of MGO to induce ER stress response.

**Figure 7:**
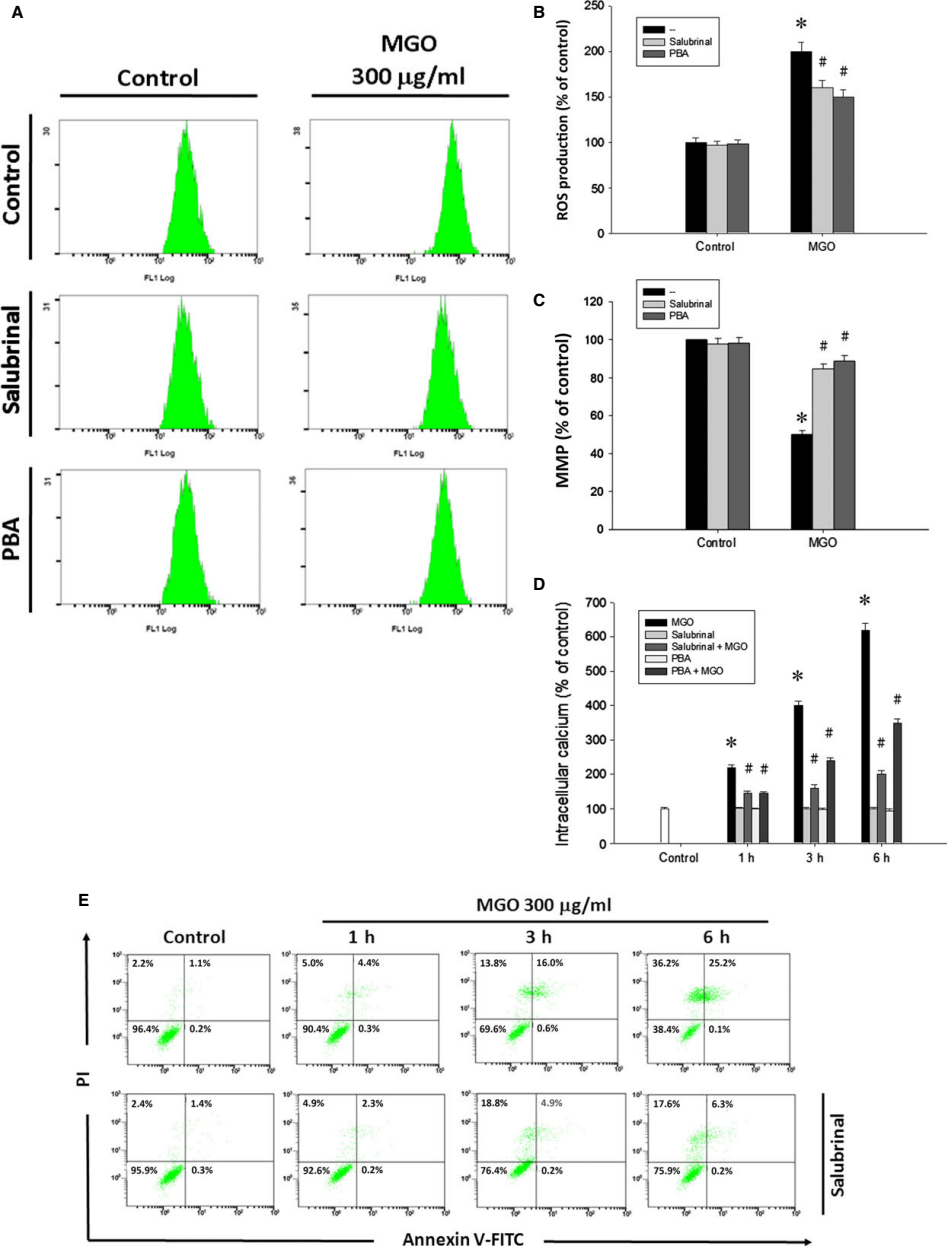
ER stress inhibitors suppress MGO‐induced ROS generation, MMP reduction, intracellular calcium increase and cell death in ARPE‐19 cells. Cells were pre‐treated with 10 μM salubrinal or 3 mM 4‐phenylbutyrate (PBA) for 1 h and then incubated with 300 μg/ml MGO. (**A, B**) After MGO incubation for 1 h, cellular ROS levels were measured with H_2_ DCFDA by using flow cytometry. (**C**) After MGO incubation for 3 h, MMP was measured with rhodamine 123 by using flow cytometry. (**D**) After MGO incubation for 3 h, intracellular calcium levels were determined by using Fluo‐3 AM and flow cytometry. *P < 0.05, indicating the significant effect of MGO alone. ^#^ *P* < 0.05, indicating the significant inhibitory effects of salubrinal and PBA on MGO responses. (**E**) After MGO incubation for 6 h, cell viability was determined by annexin V and PI staining, and evaluated by flow cytometry.

To elucidate the role of ER stress in MGO‐induced cell death, we used chemical inhibitors of ER stress, i.e. salubrinal, a selective inhibitor of eIF2α dephosphorylation 30, and 4‐PBA, a chemical chaperone of ER 31. When ARPE‐19 cells were pre‐treated with 10 μM salubrinal or 3 mM 4‐PBA for 1 h and then incubated with MGO for 1 h, the MGO‐induced ROS generation (Fig. 7A and B) as well as MMP loss (Fig. 7C) was significantly decreased. Likewise, both ER stress inhibitors can reduce the MGO‐induced intracellular calcium increase (Fig. 7D). Accordingly, salubrinal (10 μM) significantly reversed MGO‐induced cell death in ARPE‐19 cells (Fig. 7E). The viable cells were significantly increased from 38.4% to 75.9% by salubrinal. The results suggest that ER stress plays a key role in MGO‐induced ROS‐dependent cell death.

## Discussion

MGO is a physiological metabolite that is closely related to the pathogenesis in AMD and DR. Chronic hyperglycaemia induces elevated levels of intracellular production of MGO 32, and MGO may be the major source of AGEs in retina 33. Moreover, MGO is important in mediating microglial activation in the diabetic retina 34. As a highly reactive dicarbonyl metabolite, MGO can alter molecular structure and function by forming AGE‐adducts with proteins, phospholipids and nucleotides. For AGEs containing MGO, they can damage cells by three general mechanisms: as adducts occurring on modified serum proteins, as endogenous adducts formed as a consequence of glucose metabolism, or as extracellular matrix‐immobilized modifications on long‐lived structural proteins 7. MGO also plays an important role in the formation of drusen, which accumulates below RPE cells and is related to the AMD. Under the vitamin A cycling in retina, fluorescent bisretinoids, such as A2E and all‐*trans*‐retinal dimer, are formed as a by‐product and can accumulate in RPE cells as lipofuscin pigments. Further photo‐cleavage of A2E can produce reactive dicarbonyl MGO 35. Moreover, in the pathogenesis of AMD, the collagen of Bruch's membrane is increasingly cross‐linked with age 36, and MGO is a critical contributor to this covalent cross‐linking of extracellular proteins 8. Previously, MGO at the mM concentration range has been shown to affect various kinds of cellular functions such as insulin signalling 37, mitochondrial respiration and glycolysis 38. Moreover, high dose MGO therapy has been suggested for the cancer treatment 39, 40, 41. But its side effects on the normal tissue should be taken into consideration.

Apoptosis is an active physiological process to self‐destruction for maintaining cellular homeostasis between cell division and cell death. MGO has been demonstrated to induce apoptosis in several cell types 42, 43, 44, including RPE cell 45. Our study also showed that MGO decreases cell viability and induces a mixed type of cell death in ARPE‐19 cells (Fig. 1A and B). After MGO (300 μg/ml) treatment, early necrosis followed by appearance of phosphatidylserine is a unique feature, because in most examples of cell death, early apoptosis can lead to late necrosis. We currently do not have any explanations for such feature of cell death. As caspase activation is an important process in apoptosis, we showed a gradual increase of the cleaved fragments of caspase‐9, ‐3 and PARP1 by MGO treatment within 3–12 h (Fig. 1C). However, pan‐caspase inhibitor zVAD cannot reverse the cell death process (Fig. 1D and E). Therefore, we suggest that MGO‐induced cell death is independent of caspase activity; the exposure of late apoptosis marker and caspase activation are consequences of cell necrosis.

MGO has been shown to induce oxidative stress in osteoblast 44 and endothelial cells 46. Our study showed that MGO‐induced RPE cell death is through the oxidative stress coming from H_2_O_2_ rather than O_2_^−^ (Fig. 2A and B). Oxidative stress has been studied extensively in relation to the pathophysiology of AMD and is suggested to have a crucial role 47. As the location and physiological function of RPE cells, they are constantly exposed to several ROS 48. Thus, the protection of RPE cells by the antioxidants from oxidative damage is an important consideration for treating AMD. In this study, treatment with antioxidants resulted in much lower levels of MGO‐induced intracellular production of ROS. Moreover, our results from flow cytometry showed that MGO‐induced cell death in ARPE‐19 cells is greatly reduced by treatment with antioxidants (Fig. 2C–E). MGO may reduce viability *via* alterations in mitochondrial integrity 43, 44, 49. Mitochondrial integrity is crucial for cell survival, and decreased MMP is an early sign of apoptosis. Our results demonstrated that RPE cells have mitochondrial dysfunction when exposed to MGO. At higher ROS levels, longer mitochondrial permeability transition pore openings may release a ROS burst leading to destruction of mitochondria, and the released ROS can further propagate from one mitochondrion to another mitochondrion in a cell 50. Therefore, the decline in MMP caused by MGO (Fig. 3A and B) could be prevented by treating antioxidant NAC (Fig. 3C).

Elevated intracellular Ca^2+^ concentration initiates apoptosis in many cell types. Our study showed that MGO treatment causes an increase in cytoplasmic Ca^2+^ (Fig. 4A and B). SOCs are the plasma membrane Ca^2+^ channels which are a major pathway for Ca^2+^ entry under the condition of decreased Ca^2+^ content in the ER 51. Our study shows the SOC channel inhibitor can completely block the increase of the intracellular calcium (Fig. 4C). IP_3_ receptors are ligand‐gated channels that discharge Ca^2+^ from ER stores in response to stimuli 52. The release of Ca^2+^ from ER stores by IP_3_ receptors induces mitochondrial Ca^2+^ overload and cell death 53. Our results demonstrate that the IP_3_ receptor inhibitor cannot lower the increased intracellular calcium by MGO (Fig. 4C). According to some reports, ROS can induce an increase in intracellular Ca^2+^ concentration 54, 55, and calcium released from the ER augments the production of cytosolic ROS 56. In the present study, our data also support this notion. The increased intracellular Ca^2+^ by MGO could be reversed by antioxidant NAC (Fig. 5A). Besides, lowering intracellular Ca^2+^ by intracellular calcium chelator BAPTA/AM and SOC channel inhibitors can reduce the MGO‐induced ROS generation (Fig. 5B). Furthermore, decreasing intracellular Ca^2+^ by SOC channel inhibitors can reduce the MGO‐induced MMP loss (Fig. 5C).

ER stress plays a major pathological role in many ocular diseases such as retinitis pigmentosa, glaucoma and macular degeneration 57. Induction of stress by MGO in human lens epithelial cells has been demonstrated 23, but ER stress‐induced cell death by MGO in RPE cells is not known. Our studies indicate that MGO activates the initiators of typical UPR signal transduction pathways: PERK‐eIF2α‐ATF4, IRE1‐XBP1 and ATF6 (Fig. 6). In order to confirm the role of ER stress in MGO‐induced cell death, we used two different chemical ER stress inhibitors, salubrinal and 4‐PBA. The pre‐treatment with salubrinal or 4‐PBA can decrease the MGO‐induced ROS generation (Fig. 7A and B) and intracellular calcium elevation (Fig. 7D), and can reverse the MMP loss and cell death by MGO (Fig. 7C and E). These results altogether suggest that ER stress plays a key role in MGO‐induced cell death.

**Figure 8:**
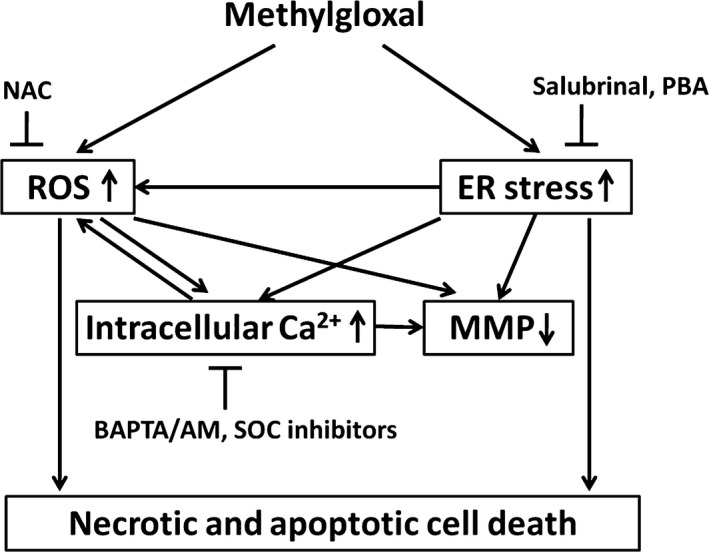
Summary of molecular mechanisms underlying MGO‐induced cell death in RPE cells.

In conclusion, we, for the first time, demonstrate that MGO can decrease RPE cell viability, resulting from the ER stress‐dependent intracellular ROS production, MMP loss and increased intracellular calcium (Fig. 8). ROS scavenger and store‐operated calcium channel inhibitors can reverse the MGO‐induced ROS production, MMP loss, intracellular calcium increase and cell death. Besides, inhibition of ER stress by salubrinal and 4‐PBA can reduce the MGO‐induced intracellular events and cell death. As MGO is one of the components of drusen in AMD and is the AGEs adduct in DR, this study could provide a valuable insight into the molecular pathogenesis of AMD and DR.

## Conflict of interest

The authors declare that they have no conflict of interest.
